# Supplementary material for: Extracellular ATP/P2X7 receptor, a regulatory axis of migration in ovarian carcinoma-derived cells
Source: PLoS One. 2024 Jun 13;19(6):e0304062. doi: 10.1371/journal.pone.0304062 (PMC11175443; doi:10.1371/journal.pone.0304062)
Supplement: S1 Fig — (PDF) [file pone.0304062.s001.pdf]

S1

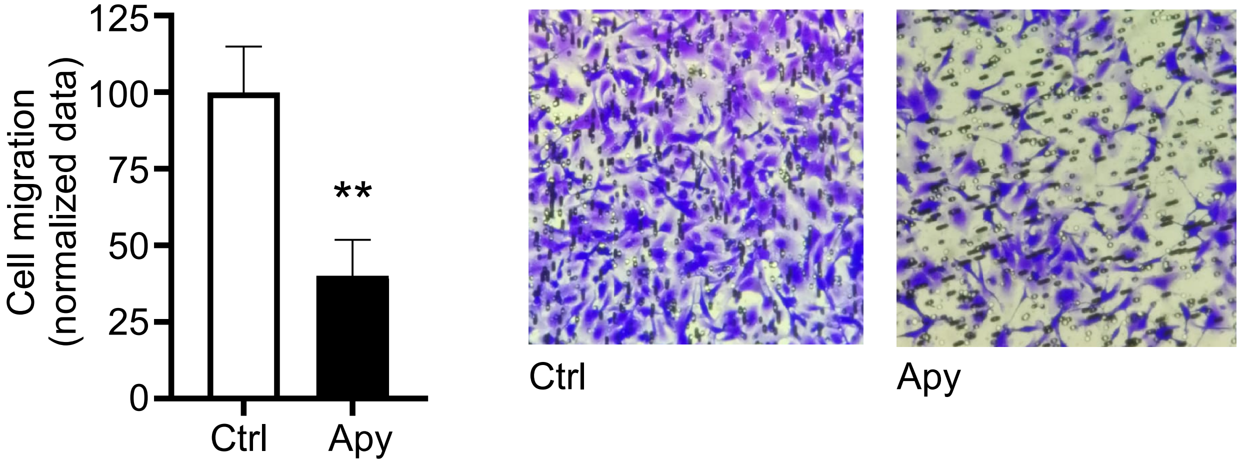

S1. ATP abolishment decreases cell migrationSKOV-3 cells were placed in the upper chamber of a transwell system, either with Apy 10 u/mL or medium alone. The upper inserts were placed inside a 24-well plate with complete medium as chemoattractant. In the graphs, bars represent the mean value ± S.E.M. of three different experiments. *$p < 0.05$.
