# Supplementary material for: Extracellular ATP/P2X7 receptor, a regulatory axis of migration in ovarian carcinoma-derived cells
Source: PLoS One. 2024 Jun 13;19(6):e0304062. doi: 10.1371/journal.pone.0304062 (PMC11175443; doi:10.1371/journal.pone.0304062)
Supplement: S2 Fig — (PDF) [file pone.0304062.s002.pdf]

S2

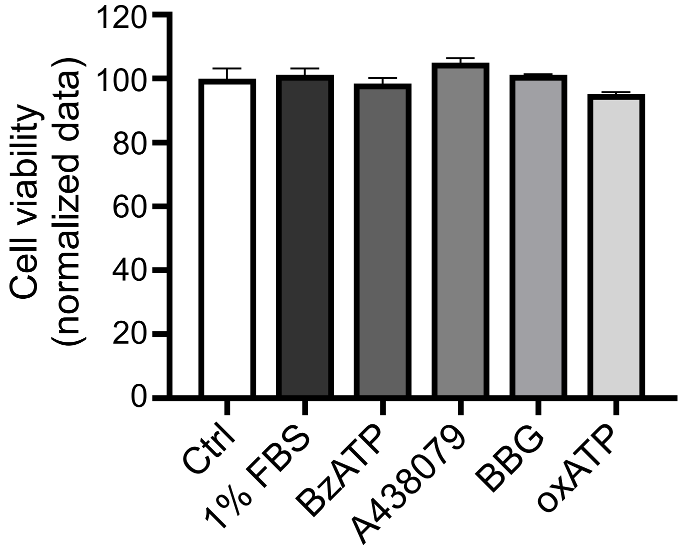

S2. P2X7 pharmacological stimulation does not affect cell viability. SKOV-3 cells were seeded at 50% confluence in 24-well plates and incubated with BzATP 50 μM, A438079 125 nM, BBG 200 nM and OxATP 200 μM. Cell viability was estimated with the MTS assay. In the graphs, bars represent the mean value ± S.E.M. Each experiment was done in sextuplicate
