# Supplementary material for: Extracellular ATP/P2X7 receptor, a regulatory axis of migration in ovarian carcinoma-derived cells
Source: PLoS One. 2024 Jun 13;19(6):e0304062. doi: 10.1371/journal.pone.0304062 (PMC11175443; doi:10.1371/journal.pone.0304062)
Supplement: S3 Fig — (PDF) [file pone.0304062.s003.pdf]

S4

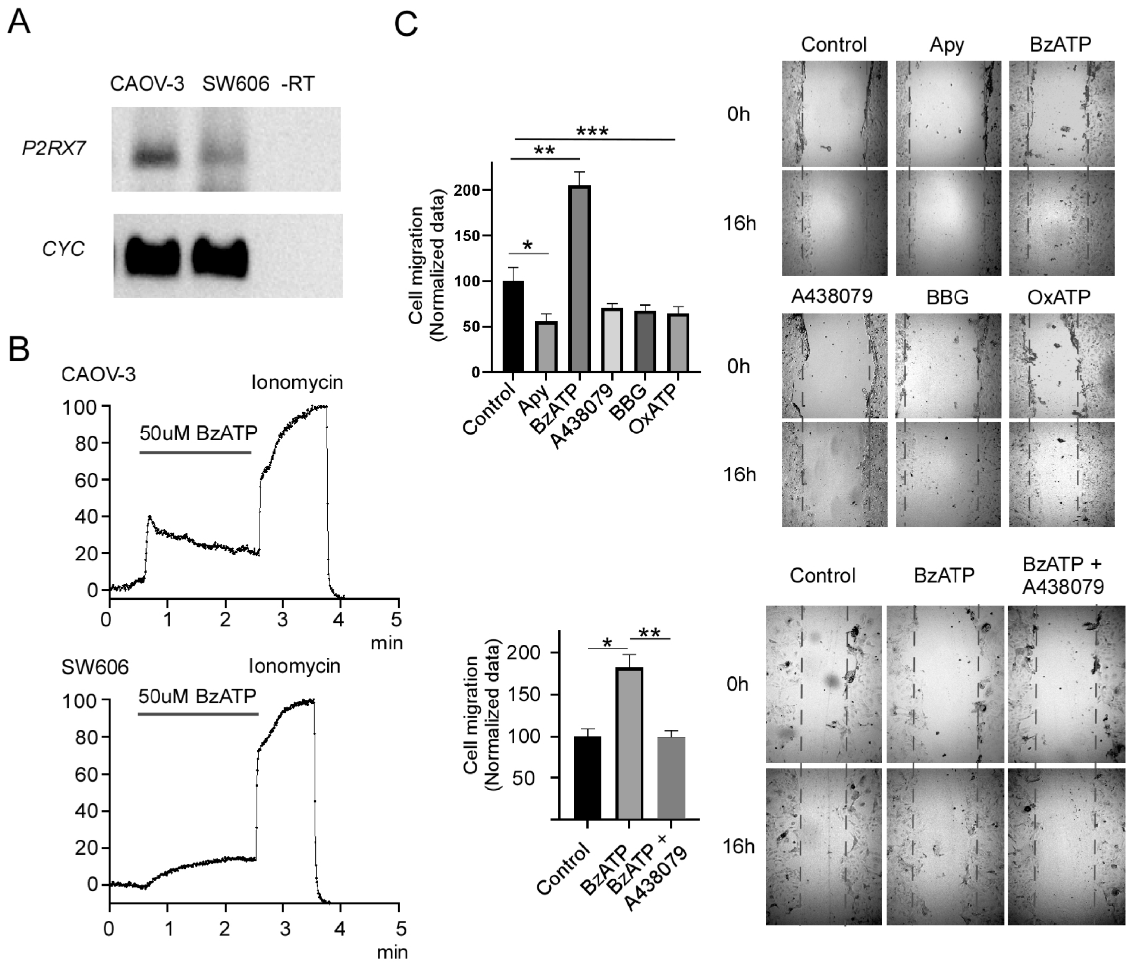

S4. A) The P2RX7 transcript was analyzed by reverse transcription followed by end-point PCR. Amplicons were analyzed in 1.5% agarose gels, purified, and sequenced to confirm their identity. B) Representative Ca2+ fluorescence (Fluo-4) traces from CAOV-3 and SW-626 cells. Cells were stimulated with 50 µM BzATP in N-Ca2+ extra-cellular solutions; at the end of the protocol, ionomycin (10µM) and MnCl2 (5mM) were sequentially applied to determine the maximum and minimum levels of intracellular Ca2+, respectively. C) Role of P2X7R in cell migration modulation measured by the wound-healing assay. Confluent cell cultures of either CAOV-3 and SW-626 were stimulated with BzATP 50 µM for 16 h, and migration was measured by the wound healing assay. A series of antagonists were also employed: the specific antagonist A438079 125 nM, non-specific antagonist BBG 200 nM, non-reversible antagonist OxATP 200 µM.
