# Supplementary material for: Extracellular ATP/P2X7 receptor, a regulatory axis of migration in ovarian carcinoma-derived cells
Source: PLoS One. 2024 Jun 13;19(6):e0304062. doi: 10.1371/journal.pone.0304062 (PMC11175443; doi:10.1371/journal.pone.0304062)
Supplement: S1 Table — (DOCX) [file pone.0304062.s004.docx]

S3 Table**.** Differentially expressed purinergic receptors in patients-derived samples

|  | *P2RY2* | | *P2RY4* | | *P2RY6* | | *P2RY11* | | *P2RX7* | |
| --- | --- | --- | --- | --- | --- | --- | --- | --- | --- | --- |
| Accession number | Log2FC | pAdjust | Log2FC | pAdjust | Log2FC | pAdjust | Log2FC | pAdjust | Log2FC | pAdjust |
| GSE222982 | -0.3299 | 1 | 0.3706 | 1 | 0.3798 | 1 | -0.1678 | 1 | 1.1518 | 6.39E-02 |
| GSE73064 | -0.1706 | 0.9994 | -2.0475 | 0.9994 | 0.9971 | 0.9994 | -0.3470 | 0.9994 | 2.0265 | 0.9994 |
| GSE73091 | -1.2943 | 1 | -2.4946 | 1 | -0.3660 | 1 | 0.1050 | 1 | 2.6936 | 0.995 |
