# Supplementary material for: Extracellular ATP/P2X7 receptor, a regulatory axis of migration in ovarian carcinoma-derived cells
Source: PLoS One. 2024 Jun 13;19(6):e0304062. doi: 10.1371/journal.pone.0304062 (PMC11175443; doi:10.1371/journal.pone.0304062)
Supplement: S2 Table — (DOCX) [file pone.0304062.s005.docx]

S5 Table. Ovarian cancer samples tested for P2X7 immunostaining in Human Protein Atlas (https://www.proteinatlas.org/ENSG00000089041-P2RX7/pathology/ovarian+cancer).

| Disease | Age | Patient ID | Staining in tumor cells |
| --- | --- | --- | --- |
| Cystadenocarcinoma serous | 59 | 2347 | Positive |
| Carcinoma endometroid | 62 | 2568 | Negative |
| Cystadenocarcinoma serous | 79 | 2889 | Positive |
| Cystadenocarcinoma serous | 79 | 2889 | Positive |
| Cystadenocarcinoma serous | 54 | 2114 | Positive |
| Cystadenocarcinoma serous | 57 | 2082 | Positive |
| Cystadenocarcinoma serous | 56 | 3115 | Positive |
| Carcinoma endometroid | 42 | 2218 | Positive |
| Cystadenocarcinoma mucinous | 73 | 1844 | Positive |
| Cystadenocarcinoma mucinous | 39 | 2950 | Positive |
| Carcinoma endometroid | 51 | 1911 | Positive |
| Cystadenocarcinoma serous | 69 | 2391 | Positive |
| Cystadenocarcinoma serous | 59 | 2347 | Negative |
| Cystadenocarcinoma serous | 56 | 3115 | Positive |
| Cystadenocarcinoma mucinous | 61 | 4369 | Negative |
| Cystadenocarcinoma mucinous | 73 | 1844 | Positive |
| Carcinoma endometroid | 42 | 2218 | Negative |
| Cystadenocarcinoma serous | 59 | 2347 | Positive |
| Cystadenocarcinoma serous | 58 | 2724 | Positive |
| Cystadenocarcinoma serous | 69 | 2391 | Positive |
| Cystadenocarcinoma serous | 54 | 2114 | Positive |
| Cystadenocarcinoma serous | 57 | 2082 | Positive |
| Cystadenocarcinoma serous | 56 | 3115 | Positive |
| Cystadenocarcinoma mucinous | 61 | 4369 | Positive |
| Cystadenocarcinoma mucinous | 39 | 4126 | Positive |
| Cystadenocarcinoma mucinous | 73 | 1844 | Positive |
| Carcinoma endometroid | 42 | 2218 | Positive |
| Carcinoma endometroid | 51 | 1911 | Positive |
| Carcinoma endometroid | 62 | 2568 | Positive |
