# Supplementary material for: Extracellular ATP/P2X7 receptor, a regulatory axis of migration in ovarian carcinoma-derived cells
Source: PLoS One. 2024 Jun 13;19(6):e0304062. doi: 10.1371/journal.pone.0304062 (PMC11175443; doi:10.1371/journal.pone.0304062)

Fig. 1, C).

Antibody to E-cadherin (1:1000) (Cell Signaling 144725).

Order: Apyrase 10u/mL I /Control I /Apyrase 10umL II / Control II / Apyrase III / Control III

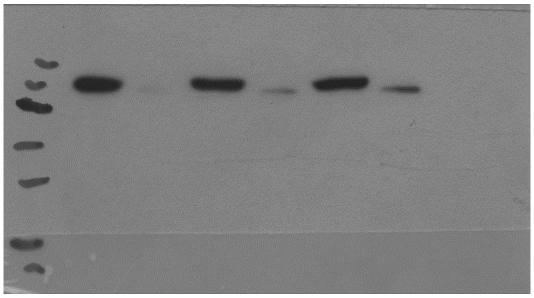

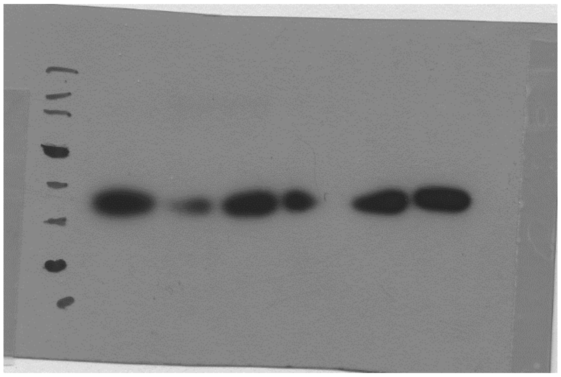

Antibody to GAPDH (1:1000) (Cell Signaling 2118).

Order: Apyrase 10u/mL I /Control I /Apyrase 10umL II / Control II / Apyrase III / Control III

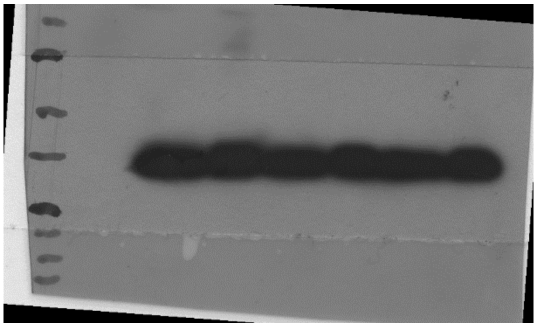

Antibody to Vimentin (1:1000) (Cell Signaling 3932).

Order: Apyrase 10u/mL I /Control I /Apyrase 10umL II / Control II / Apyrase III / Control III

Fig. 6, C).

Antibody to E-cadherin (1:1000) (Cell Signaling 144725).

Samples set I.

Order: Control, BzATP 50µM, A438079 125nM, Brilliant Blue G 200nM, Oxidized ATP 200 µM.

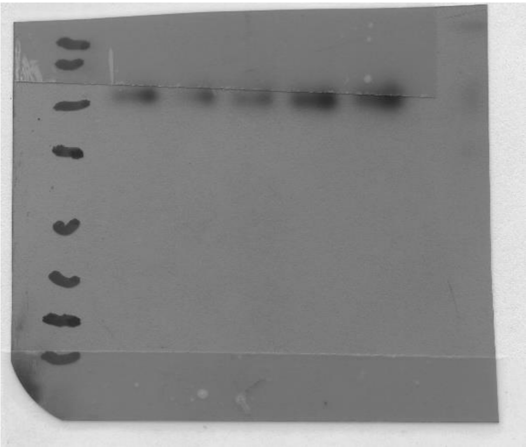

Samples set II.

Order: Control, BzATP 50µM, A438079 125nM, Brilliant Blue G 200nM, Oxidized ATP 200 µM.

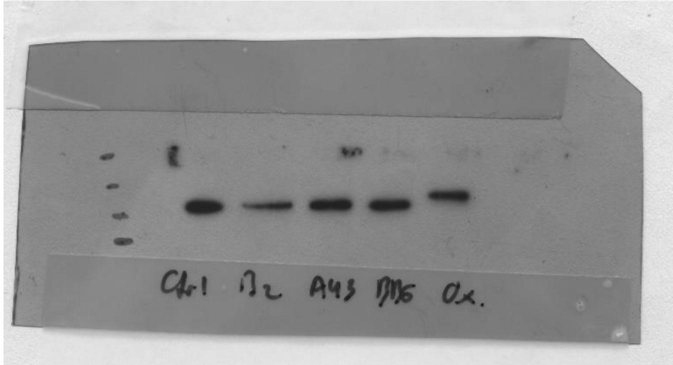

Samples set III.

Order: Control, BzATP 50µM, A438079 125nM, Brilliant Blue G 200nM, Oxidized ATP 200 µM.

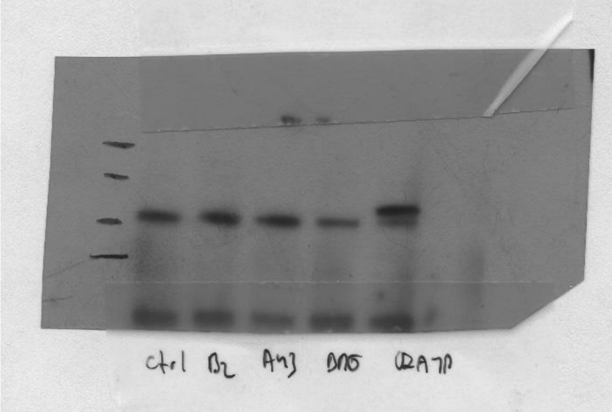

Antibody to β-actin (1:1000) (Sigma A2066).

Samples set I.

Order: Control, BzATP 50µM, A438079 125nM, Brilliant Blue G 200nM, Oxidized ATP 200 µM.

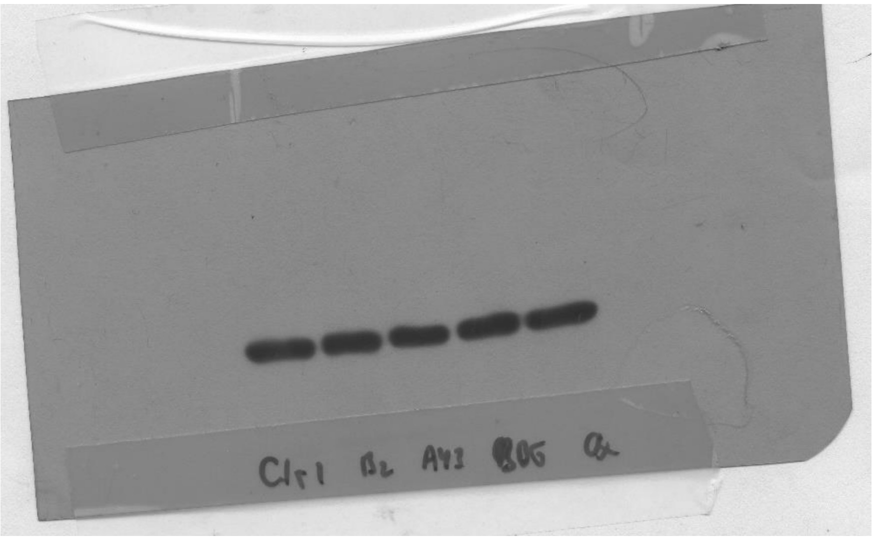

Samples set II.

Order: Control, BzATP 50µM, A438079 125nM, Brilliant Blue G 200nM, Oxidized ATP 200 µM.

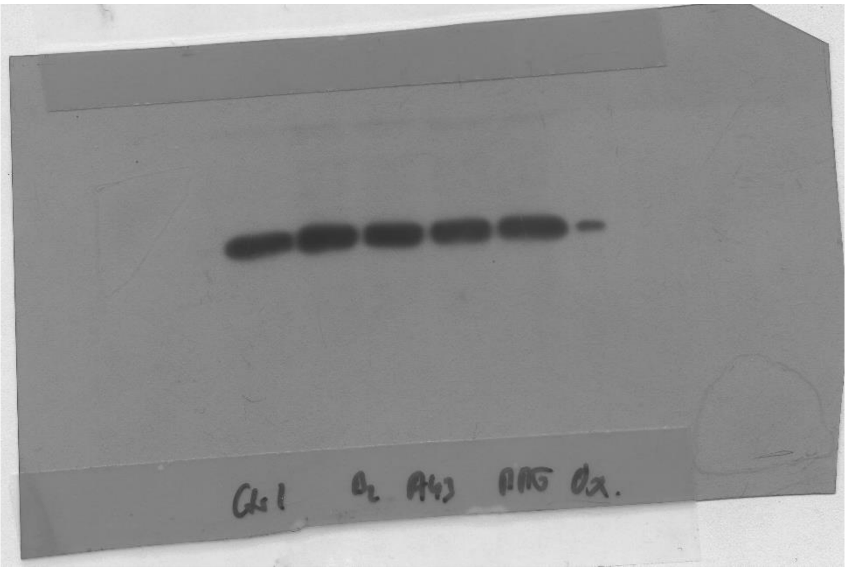

Samples set III.

Order: Control, BzATP 50µM, A438079 125nM, Brilliant Blue G 200nM, Oxidized ATP 200 µM.

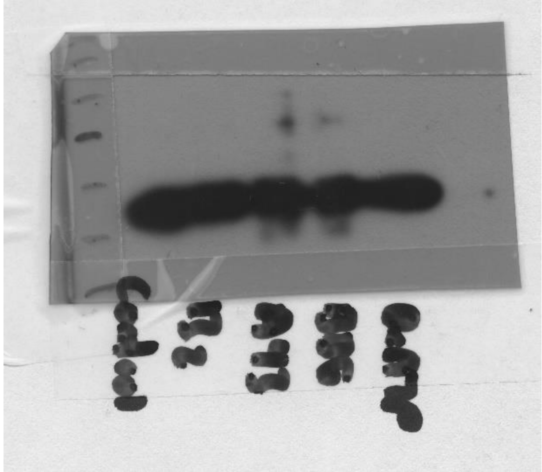

Supplement: S1 Raw images — (PDF) [file pone.0304062.s006.pdf]
